# Emotional Valence Influences Empathic Capacity in Adolescence and Young Adulthood

**DOI:** 10.1002/jad.70216

**Published:** 2026-07-06

**Authors:** Alana J. Topsfield, Sarah A. Grainger

**Affiliations:** ^1^ The University of Queensland Brisbane Queensland Australia

**Keywords:** affective empathy, cognitive empathy, ecological validity, valence

## Abstract

**Introduction:**

Previous research has identified age‐related differences in empathic abilities between adolescence and young adulthood. While cognitive empathy appears to continue to develop from adolescence and into adulthood, the developmental trajectory of affective empathy is less clear. It is also unclear how valence influences empathic responding and whether this differs according to age. To address this, we assessed age differences in cognitive and affective empathy in adolescents and young adults using a newly developed empathy measure that includes contextually rich stimuli with a variety of positive and negative emotions.

**Methods:**

We recruited 109 participants, including 50 adolescents (aged 13–20 years, 60% female) and 59 young adults (aged 25–35 years, 59% female). All participants completed the EMOTE, which indexed cognitive empathy accuracy and affective empathy ratings. All data were collected in Australia between 2024 and 2025.

**Results:**

Adolescents had lower cognitive empathy accuracy than young adults, whereas affective empathy ratings did not differ between the age groups. Emotional valence influenced empathic responding, such that cognitive empathy accuracy was higher for positive compared with negative stimuli in both age groups, and affective empathy ratings were stronger for negative compared with positive stimuli in adolescents only.

**Conclusion:**

Our findings support the view that cognitive empathy continues to develop into young adulthood, while affective empathy remains more stable. Critically, our results highlight the role of emotional valence in shaping empathic responding, particularly in adolescence.

## 1 Introduction

Empathy—the capacity to understand and feel the emotions of others—is a core social cognitive skill that supports the development and maintenance of positive social relationships. It is generally accepted that empathy is multidimensional, encompassing both cognitive and affective components (de Waal and Preston 2017; Decety and Cowell 2014; Deutsch and Madle 1975; Shamay‐Tsoory 2011). While cognitive empathy refers to the ability to understand and interpret the emotional and mental states of others, affective empathy refers to one's emotional response to another person's emotional experience.

While empathy plays a crucial role in social functioning at all stages of the lifespan, it is particularly important in adolescence. This is because adolescence is an important developmental period characterized by a process of social reorientation (Nelson et al. 2005; Nelson et al. 2016) where establishing relationships with peers is crucial for broader wellbeing (Mitic et al. 2021; Truong and Joshi 2024). Indeed, several studies have found that higher levels of empathy in adolescence are linked to important social outcomes, such as increased friendship quality (Chow et al. 2013; Ciarrochi et al. 2017; Meuwese et al. 2017; Overgaauw et al. 2017) and prosocial behavior (Li et al. 2025). Further, lower levels of empathy, particularly affective empathy, have been linked to bullying behavior and externalizing problems (Morosan et al. 2020; van Noorden et al. 2015).

Several studies have now shown that cognitive empathy continues to develop throughout adolescence into adulthood, whereas age‐differences in affective empathy are less clear (Dorris et al. 2022; Eisenberg et al. 2005; Gaspar and Esteves 2022; Kim et al. 2020; Kunzmann et al. 2018; Tremblay et al. 2022; Tousignant et al. 2017; Vetter et al. 2013). However, this literature has relied primarily on self‐report questionnaires (e.g., Eisenberg et al. 2005; Tremblay et al. 2022; Tousignant et al. 2017). While self‐report measures are convenient to administer, they rely on respondents’ metacognitive insight into their own empathic abilities, and are also susceptible to social desirability biases, which can influence their validity. Given that metacognitive abilities are still developing in adolescence (Weil et al. 2013) and that adolescent behaviors are strongly motivated by social acceptance (Blakemore and Mills 2014), empathy measurement type is a particularly important consideration in adolescent studies.

Behavioral or performance‐based measures of empathy provide a more objective assessment and therefore may provide a more accurate index of adolescents’ true empathic abilities. These include tasks such as the Reading the Mind in the Eyes test (RMET; Baron‐Cohen et al. 2001) and the Dynamic Emotional Expression Recognition Task (DEER‐T; Platt et al. 2010), which require detecting emotion from either static images or videos of morphing images. However, relatively few studies have used such tasks to compare performance across adolescents and young adults. Additionally, these studies have typically used behavioral tasks lacking ecological validity. That is, they use tasks that fail to present social and emotional information as it is typically encountered in daily life. This is particularly problematic in the context of adolescent empathic development due to adolescents’ heightened sensitivity to social information (Blakemore and Mills 2014).

Indeed, nearly all behavioral cognitive empathy studies comparing adolescents and young adults have required detecting emotion from static images (e.g., Dorris et al. 2022; Kim et al. 2020; Tousignant et al. 2017; Vetter et al. 2013). While such tasks do provide a more objective measure of empathy compared with self‐reports, they do not include the dynamic expressions, body movements and vocalizations that are present in real‐life social situations (Buck et al. 2017; Piwek et al. 2015; Reed et al. 2020; Richoz et al. 2018). There are only two studies to our knowledge that used multimodal tasks to investigate empathy in adolescents and adults. Kunzmann et al. (2018) presented adolescents and adults with videos of people recalling emotional autobiographical memories. While they did not assess affective empathy, they found age effects in cognitive empathy, such that adolescents performed worse than young adults. Another study, Breil et al. (2021), developed a version of the “EmpaToM” (Kanske et al. 2015) to measure cognitive theory of mind (ToM) and affective empathy in adolescence: the EmpaToM‐Y. This task includes videos of paid actors describing neutral or emotionally negative stories to a camera. The EmpaToM‐Y was initially validated in young adults and subsequently tested for feasibility in adolescents; however, the study design did not allow for direct comparison between these age groups. While both Kunzmann et al. (2018) and Breil et al. (2021) used dynamic tasks with enhanced ecological validity, the stimuli lacked rich contextual information. This is because the stimuli were filmed in standardized laboratory conditions and involved a single person speaking to a camera. It is well established that context plays a key role in emotion perception (Aviezer et al. 2008; Aviezer et al. 2012; Aviezer et al. 2017; Barrett et al. 2011). Indeed, prior work has shown that context is particularly beneficial for older adults’ emotion recognition abilities, with age differences eliminated when congruent contextual cues are provided (Noh and Isaacowitz 2013). However, it remains unclear whether adolescent empathic performance differs when contextually rich stimuli are used.

A further problem is that both prior studies using dynamic tasks focussed on a small range of predominantly negative emotions, offering limited insight into potential effects of valence. Indeed, Kunzmann et al. (2018) included stimuli depicting only three emotions (sadness, anger, and happiness) and Breil et al. (2021) included only neutral and negative stimuli. While the authors of the latter study primarily included valence in their analysis as a manipulation check, Kunzmann et al. (2018) found that empathic accuracy was higher when happiness was the dominant emotion relative to when anger or sadness were the dominant emotion. However, happiness was the only positive emotion and therefore may have been easier to detect when presented amongst two clearly negative emotions. Nevertheless, this finding suggests age differences in empathic accuracy may differ according to valence, warranting further investigation with a larger range of positive and negative emotions.

The current study aimed to address these gaps by assessing cognitive and affective empathy in a sample of adolescents and young adults using a newly developed empathy measure with enhanced ecological validity: the EMOTE (Grainger et al. 2025). This task includes contextually rich footage of real people experiencing genuine emotions as they unfold “in the moment” and includes a broad range of positive and negative emotions. Consistent with prior work (e.g., Dorris et al. 2022; Gaspar and Esteves 2022; Kunzmann et al. 2018; Tremblay et al. 2022; Vetter et al. 2013), we predicted that adolescents would show lower levels of cognitive empathy accuracy relative to young adults. While there are mixed findings as to whether there are age effects in affective empathy (Gaspar and Esteves 2022; Kim et al. 2020; Tremblay et al. 2022), the enhanced ecological validity of the EMOTE may elicit strong empathic responses in both groups and therefore no age differences were expected for affective empathy. Given the limited evidence regarding valence effects, we did not make any a priori predictions for valence.

## 2 Methods

We pre‐registered our study design and hypotheses on the Open Science Framework (https://osf.io/u9t3s/overview?view_only=be555375564043f0bb41d654e1488d3f).

### 2.1 Participants

An a priori power analysis revealed that 84 participants (42 in each age group) were required to detect a moderate effect of age‐group with 80% power. This was based on Kunzmann et al. (2018) who found a moderate‐sized effect of age on empathic accuracy (*d* = 0.54). We ended up exceeding this with 109 participants in the final sample: 50 adolescents (13–20 years, *M* = 15.80, SD = 2.00) and 59 young adults (25–35 years, *M* = 28.86, SD = 3.20). The age range of adolescence was selected to align with both traditional definitions of adolescence (13–18 years) and more recent definitions which extend adolescence into the early twenties (Sawyer et al. 2018). Young adulthood was therefore defined as beginning at 25 years to ensure clear developmental separation between the age groups. Gender was consistent across the age groups, with approximately 60% of adolescents and young adults identifying as female. Adolescent participants were predominantly recruited through existing partnerships with independent Brisbane schools and word of mouth, while young adults were mostly recruited from the local community. Nine adolescents and eight young adults were also recruited from the University of Queensland (UQ) first‐year psychology research participation scheme (SONA). Participants self‐identified as Caucasian (56.9%), Asian (27.5%), Indian (5.5%), or Other (10.1%). A chi‐square test of independence indicated that ethnicity did not significantly differ between the age groups, *χ* ^2^(3) = 6.92, *p* = 0.074. All participants were fluent in English and not currently experiencing any learning disorders or neurodevelopmental, neurological, or psychiatric conditions. The UQ Human Research Ethics Committee provided ethical clearance for this study (Approval number: 2024/HE000374).

### 2.2 Measures

#### 2.2.1 The EMOTE

The EMOTE (Grainger et al. 2025) was included as a task‐based measure of cognitive and affective empathy. Stimuli consist of 40 multimodal videos of people experiencing genuine emotions as they unfold “in the moment.” These videos are sourced primarily from YouTube and are edited to be no longer than 90 s in length. All videos include spoken dialog and do not include any artificial background (e.g., green screen) or music. The videos include a range of protagonists with different demographic characteristics (i.e., varying in age, gender, and race/ethnicity). Across the stimuli set, 20 videos are negatively valenced, and 20 are positively valenced, with 14 different negative (e.g., distressed, frustrated, terrified) and 14 different positive emotions (e.g., excited, proud, amused) included in the set. The full list of emotions can be found on the OSF (https://osf.io/nadr6/overview). All videos in this task were presented in a randomized order and there was no time limit to make responses. Cognitive empathy is assessed by asking participants to identify the dominant emotion the protagonist was feeling from a list of four emotions (one target and three distractor emotions). Overall accuracy scores for cognitive empathy are determined by calculating the percentage of trials where a participant correctly identifies the target emotion. Affective empathy is assessed by asking participants to rate their emotional response to the video, where participants respond to the question, “To what extent do you feel what the main person is feeling?” on a scale from 1 (not at all) to 9 (very much). Affective empathy ratings are averaged to create a mean affective empathy score. The EMOTE takes approximately 40 min to complete.

### 2.3 Procedure

Data for this study was collected as part of a larger project investigating social cognition in adolescence and young adulthood. Parents/guardians of school students aged 13–18 who had previously participated in research at UQ were sent study information, and interested parents were provided with a link to sign up and provide informed parental consent online. Young adults were primarily recruited through physical flyers around the local community and social media advertisements, and undergraduate psychology students signed up through the SONA system. Participants attended an in‐person session in our research laboratory and were given an information sheet and asked to provide informed consent upon arrival. Participants then completed the EMOTE as well as other tasks that are not relevant to this study and therefore not reported here. Participants also completed demographic and self‐report questionnaires, including measures of social functioning (i.e., social anxiety and social connectedness) which were included as exploratory variables (see the Material S1 for more details). Relationships between these measures and empathy in adolescence and young adulthood can be seen in Tables 2 and 3, respectively. All measures were presented in counterbalanced blocks through the Qualtrics survey software. Upon study completion, participants were provided with a written debrief and thanked for their involvement. SONA participants were assigned 3 course credits and paid participants received a $50 EFTPOS gift card.

**Table 2:** Pearson correlations between age, gender, cognitive and affective empathy scores, social anxiety, and social connectedness in adolescents.

Variable	1	2	3	4	5	6	7	8	9	10
1. Age	–									
2. Gender	0.02	–								
3. Cognitive Empathy Total	0.08	−0.01	–							
4. Cognitive Empathy Negative	0.18	−0.11	0.82**	–						
5. Cognitive Empathy Positive	−0.09	0.13	0.67**	0.12	–					
6. Affective Empathy Total	0.27	−0.24	0.09	0.09	0.04	–				
7. Affective Empathy Negative	0.26	−0.23	0.12	0.11	0.07	0.99**	–			
8. Affective Empathy Positive	−0.24	−0.24	0.06	0.07	0.00	0.99**	0.94**	–		
9. Social Connectedness	−0.01	−0.21	0.05	0.10	−0.03	0.13	0.11	0.14	–	
10. Social Anxiety	−0.11	0.33*	−0.06	−0.13	0.07	−0.29*	−0.28	−0.30*	−0.78**	–

*Note:* Male = 1, Female = 2.

* *p* < 0.05

** *p* < 0.01.

**Table 3:** Pearson correlations between cognitive and affective empathy scores, social anxiety, and social connectedness in young adults.

Variable	1	2	3	4	5	6	7	8	9	10
1. Age	–									
2. Gender	0.24	–								
3. Cognitive Empathy Total	−0.17	−0.07	–							
4. Cognitive Empathy Negative	−0.14	−0.15	0.80**	–						
5. Cognitive Empathy Positive	−0.13	0.05	0.80**	0.28*	–					
6. Affective Empathy Total	0.20	0.33*	0.02	0.00	0.04	–				
7. Affective Empathy Negative	0.18	0.33*	0.03	−0.01	0.05	0.98**	–			
8. Affective Empathy Positive	0.22	0.31*	0.02	0.00	0.03	0.98**	0.94**	–		
9. Social Connectedness	0.07	0.31*	0.22	0.15	0.20	0.21	0.18	0.23	–	
10. Social Anxiety	0.08	0.07	−0.21	−0.18	−0.17	0.29*	0.30*	0.28*	−0.36	–

*Note:* Male = 1, Female = 2.

* *p* < 0.05

** *p* < 0.01.

### 2.4 Analysis Plan

The central research question was addressed through two separate 2 (age‐group: adolescent, young adult) × 2 (valence: positive, negative) mixed ANOVAs, with age‐group as the between‐subjects factor and valence as the within‐subjects factor. Cognitive empathy accuracy and affective empathy ratings served as the dependent variables.

## 3 Results

Full descriptive statistics for cognitive empathy accuracy and affective empathy ratings for both positive and negative stimuli are reported in Table 1. Correlational analyses between individual difference variables and empathy are presented in Tables 2 and 3. Several significant associations were observed. Notably, gender was associated with affective empathy in adults but not adolescents, whereby being female was associated with higher levels of affective empathy. Additionally, social anxiety was associated with gender in the adolescent group only, whereby adolescent females appeared to report higher levels of social anxiety than males. Finally, social anxiety was negatively correlated with affective empathy in adolescents but positively correlated with affective empathy in adults.

**Table 1:** Descriptive statistics for cognitive empathy accuracy and affective empathy ratings.

	Adolescents	Young adults	Full sample
	*M* (SD)	*M* (SD)	*M* (SD)
Cognitive Empathy Accuracy (%)	66.68 (9.29)	71.55 (8.20)	69.32 (9.01)
Negative	61.71 (13.97)	67.06 (10.28)	64.60 (12.35)
Positive	71.63 (10.69)	76.02 (10.29)	74.01 (10.66)
Affective Empathy Rating (1–9)	5.82 (1.60)	5.83 (1.62)	5.83 (1.61)
Negative	5.94 (1.63)	5.83 (1.65)	5.89 (1.63)
Positive	5.68 (1.62)	5.83 (1.65)	5.76 (1.63)

*Note: N* = 109, *n* = 50 for adolescent group, *n* = 59 for young adult group.

### 3.1 Cognitive Empathy

A mixed ANOVA with cognitive empathy accuracy as the dependent variable, age‐group (adolescent, young adult) as the between‐subjects factor and valence (positive, negative) as the within‐subjects factor revealed a significant main effect of age‐group on cognitive empathy, *F*(1, 107) = 8.40, *p* = 0.005, *η*_p_^2^ = 0.07, such that adolescents had lower cognitive empathy (*M* = 66.68, SD = 9.29) than young adults (*M* = 71.55, SD = 8.20) (see Figure 1a). As can be seen in Figure 1b, there was also a significant main effect of valence on cognitive empathy, *F*(1, 107) = 46.50, *p* < 0.001, *η*_p_^2^ = 0.30, such that participants had lower cognitive empathy for negative (*M* = 64.60, SD = 12.35) than positive stimuli (*M* = 74.01, SD = 10.66). However, there was no significant interaction between age‐group and valence for cognitive empathy, *F*(1, 107) = 0.12, *p* = 0.727, *η*_p_^2^ = 0.001.

**Figure 1:**
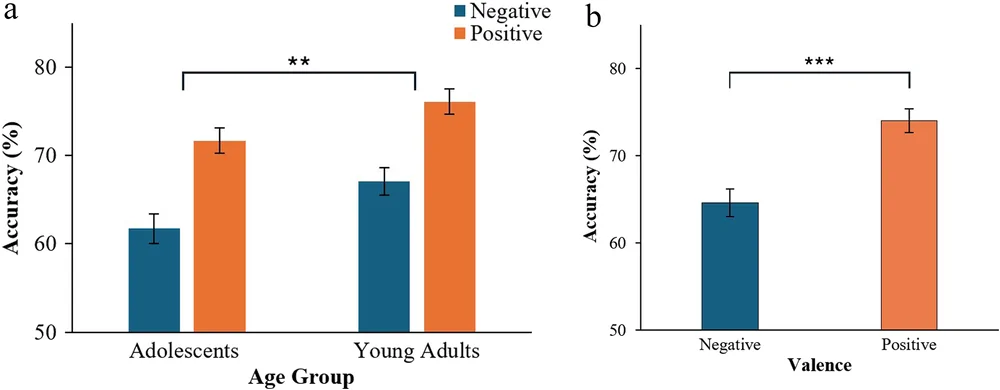
(a) Cognitive empathy accuracy by age‐group with main effect of age‐group. *Note:* Error bars represent standard error. **p < 0.01. (b) Cognitive empathy accuracy by valence, collapsed across age group, with main effect of valence. *Note:* Error bars represent standard error. ***p < 0.001.

### 3.2 Affective Empathy

A second mixed ANOVA with affective empathy ratings as the dependent variable, age‐group (adolescent, young adult) as the between‐subjects factor and valence (positive, negative) as the within‐subjects factor revealed that there was no significant effect of age‐group on affective empathy ratings, *F*(1, 107) = 0.00, *p* = 0.951, *η*_p_^2^ = 0.00. However, there was again a significant main effect of valence, *F*(1, 107) = 5.84, *p* = 0.017, *η*_p_^2^ = 0.05, such that participants had higher affective empathy for negative (*M* = 5.89, SD = 1.63) than positive stimuli (*M* = 5.76, SD = 1.63). Importantly, there was also a significant interaction between age‐group and valence, *F*(1, 107) = 5.91, *p* = 0.017, *η*_p_^2^ = 0.05. This was followed up with paired samples *t*‐tests which revealed that adolescents had higher affective empathy for negative (*M* = 5.94, SD = 1.63) than positive stimuli (*M* = 5.68, SD = 1.62), *t*(49) = 3.42, *p* < 0.001, *d*_*z*_ = 0.48, whereas young adults did not significantly differ in their affective empathy for positive (*M* = 5.83, SD = 1.65) and negative stimuli (*M* = 5.83, SD = 1.65). These data are presented graphically in Figure 2.

**Figure 2:**
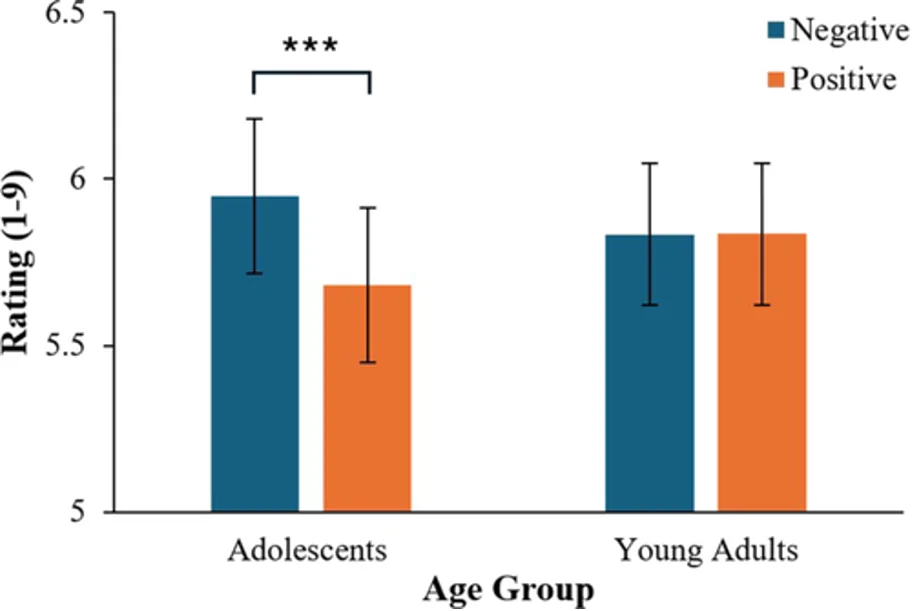
Mean affective empathy ratings by age‐group with a significant interaction between age‐group and valence. *Note:* Error bars represent standard error. ***p < 0.001.

## 4 Discussion

The primary aim of this study was to examine age‐related differences in empathy in adolescents and young adults using the EMOTE: a novel measure of cognitive and affective empathy with enhanced ecological validity. Specifically, we sought to examine whether previous findings about age differences in cognitive empathy also emerged in response to stimuli containing contextually rich, authentic emotional information, and to clarify the mixed results regarding age differences in affective empathy. We also aimed to investigate whether there were any effects of valence, and whether any valence effects varied according to age‐group.

As predicted, adolescents showed lower levels of cognitive empathy compared with young adults, suggesting ongoing development across this period. This is consistent with findings from previous studies that used self‐report and behavioral empathy tasks (Dorris et al. 2022; Gaspar and Esteves 2022; Tremblay et al. 2022; Vetter et al. 2013), including Kunzmann et al. (2018) who similarly identified a moderate‐sized age effect with dynamic video stimuli. These findings thereby extend this literature by demonstrating that adolescents show lower cognitive empathy compared with young adults to a similar degree, even when provided with emotional stimuli containing rich contextual information.

Consistent with prior work (Gaspar and Esteves 2022; Kunzmann et al. 2018), our findings also revealed a large effect of emotional valence on cognitive empathy, suggesting a substantial difference in cognitive empathy responding for positive compared with negative emotions. While Kunzmann et al. (2018) also found large effects of valence on empathic accuracy, such that empathic accuracy was increased for positive compared with negative emotions, their paradigm included only three emotions, with happiness being the only positive emotion. A large effect of emotional valence was also found on a static empathy task (Gaspar and Esteves 2022), although again, this study also only included one positive emotion. Our findings thereby meaningfully extend this work by showing that this valence effect is both meaningful and robust, with cognitive empathy accuracy for positive emotions being higher than negative emotions for both age groups, even when a broad range of positive and negative emotions are included. This greater accuracy for positive emotions may be due to the fact that they are encountered more frequently than negative emotions in everyday life (Calvo et al. 2014; Somerville and Whalen 2006) and tend to be the most stereotypical and predictable emotional expressions, making them easier to identify (Gaspar et al. 2014). Negative emotions on the other hand are perceived as being more complex, as they convey richer social information and therefore require greater cognitive processing to interpret (Fischer and Manstead 2008; Vaish et al. 2008). Moreover, negative stimuli are generally perceived as being more differentiated than positive stimuli, and consequently there is more variation in the vocabulary used to describe negative stimuli, making them more difficult to decode (Rozin and Royzman 2001).

Another key finding was that adolescents and young adults did not differ in their overall capacity for affective empathy. While the current literature is mixed, this finding is consistent with some prior research showing that adolescents and young adults self‐report similar affective empathy scores (Gaspar and Esteves 2022; Tremblay et al. 2022), and perform similarly on behavioral tasks (Kim et al. 2020). However, we again found that affective empathy was influenced by emotional valence, with participants reporting stronger affective empathy for negative compared with positive emotions, although this effect was small in magnitude. Critically though, this was qualified by a significant interaction between valence and age‐group, with a moderate effect size, which indicated that adolescents, but not young adults, showed greater affective empathy for negative relative to positive emotions. This may be interpreted through the well documented negativity bias in emotional processing, which posits that negative events elicit stronger responses than positive events of comparable intensity (Norris 2021; Rozin and Royzman 2001). Indeed, adolescents are theorized to display an increased sensitivity to social information (Blakemore and Mills 2014), and heightened affective responses to negative social interactions in particular (Sebastian et al. 2010). These findings therefore show for the first time that age differences in affective empathy depend on emotional valence, highlighting the importance of considering valence when studying empathic responding in adolescence.

Our exploratory analyses indicated that gender was correlated with affective empathy in adults but not adolescents. Specifically, being female was associated with higher affective empathy ratings in the adult group. These results are consistent with a large body of prior work showing that adult females typically report greater affective empathy than males (McDonald and Kanske 2023; Rajasekhar et al. 2025; Toussaint and Webb 2005). While gender differences in affective empathy have also been reported in adolescence, the evidence is more mixed (Michalska et al. 2013; Stuijfzand et al. 2016; Yavuz et al. 2024), with some studies suggesting that these effects may differ from early to late adolescence and other studies reporting no gender effects. Additionally, the adolescent group in our study had a relatively wide age range, which may have reduced sensitivity to detect these more nuanced effects. It is important to note that these correlations were exploratory and relied on relatively small samples and thus should be interpreted with caution.

This study provided the first assessment of adolescent empathic responding using contextually rich emotion stimuli with a broad range of emotions, providing novel insights into age‐group and valence effects. However, several potential limitations should be noted with respect to the sample and study design. Firstly, although our sample size (*N* = 109) was sufficiently powered to detect moderate sized age differences, it was underpowered to detect more subtle age differences. While small age differences may have limited practical significance, future studies would benefit from recruiting larger samples to test whether more nuanced age‐related variation in empathic responding exists. Secondly, the use of relatively wide‐ranging age groups may have reduced sensitivity to detect variability within each age group. Third, the sample was predominantly WEIRD (Western, Educated, Industrialized, Rich, and Democratic) and therefore findings may not generalize to other demographics. Future research should incorporate more diverse samples to determine whether these effects are also observed in other populations. Fourth, the cross‐sectional study design constrains conclusions about true developmental differences in empathy. Indeed, longitudinal designs that follow the same participants over time are needed to more directly examine how empathy develops throughout adolescence into young adulthood.

In addition to sample and design considerations, there are measurement‐related limitations that should be acknowledged. Firstly, affective empathy for positive and negative emotions was highly correlated in both age groups. While a significant interaction between valence and age group was observed, suggesting meaningful variation in empathic responding, the limited differentiation between valence conditions may have reduced sensitivity to valence‐specific effects on affective empathy. Secondly, while cognitive empathy responding was performance‐based (i.e., accuracy), affective empathy relied on self‐report ratings and therefore may have been influenced by response biases such as social desirability, as well as limits on metacognitive insight. Future research would benefit from adopting multi‐method approaches to measuring affective empathy, including physiological measures, to help mitigate the limitations inherent to self‐report measures. Finally, it should be noted that we did not account for the familiarity of topics discussed in our stimuli. Given adolescents’ heightened sensitivity to social information, especially that which is related to peers (Blakemore and Mills 2014; Somerville 2013), it is possible that adolescents respond differently to emotional scenarios involving adolescent targets, compared with children or adults. While the EMOTE includes targets of all ages, and previous research has found that including familiarity as a covariate does not influence the pattern of findings for cognitive empathy (Kunzmann et al. 2018), it is unclear how this may affect affective empathy. Therefore, future research should examine whether familiarity with emotional topics influences age‐related differences in affective empathy, using tasks that involve stimuli highly relevant to adolescents.

## 5 Conclusion

Taken together, our findings indicate that age‐related differences in empathy differ according to the specific empathy domain being assessed and the emotional valence of the stimuli. Adolescents demonstrated lower cognitive empathy compared with young adults, whereas affective empathy did not differ between the age groups. Importantly, we showed for the first time that emotional valence differentially influenced cognitive and affective empathy, with cognitive empathy being facilitated for positive emotions but affective empathy being stronger for negative emotions, particularly in adolescence. These results provide further support for the view that cognitive empathy continues to develop into young adulthood and highlights the importance of considering emotional valence when investigating affective emotional responses in this developmental period. An important next step in this literature will be incorporating tasks with strong ecological validity into longitudinal designs to precisely track the developmental trajectory of cognitive and affective empathy throughout adolescence and into adulthood.

## Author Contributions

**Alana J. Topsfield:** writing – original draft, formal analysis, data curation, project administration, visualization, conceptualization, investigation, methodology. **Sarah A. Grainger:** conceptualization, investigation, funding acquisition, methodology, supervision, resources, writing – review and editing.

## Conflicts of Interest

The authors declare no conflicts of interest.

## Supporting information

Supporting File

## Data Availability

The data that support the findings of this study are available from the corresponding author upon reasonable request.
