# Supplementary material for: Emotional Valence Influences Empathic Capacity in Adolescence and Young Adulthood
Source: J Adolesc. 2026 Jul 6;98(7):2330–7. doi: 10.1002/jad.70216 (PMC13643624; doi:10.1002/jad.70216)
Supplement: Supplementary file 1 [file JAD-98-2330-s001.docx]

**Supplementary material to**

Emotional valence influences empathic capacity in adolescence and young adulthood

Alana J. Topsfield^1^ & Sarah A. Grainger^1^

^1^The University of Queensland

**Self-report Questionnaires and Correlations with Empathy**

We included several self-report measures of social functioning, specifically social anxiety and social connectedness, to investigate how they may have been related to cognitive and affective empathy in adolescence and young adulthood. Relationships between these measures and empathy in adolescence can be seen in Table 2, and young adults in Table 3 in the main text. We describe these in more detail below.

**Measures**

***Liebowitz Social Anxiety Scale***

The Liebowitz Social Anxiety Scale (LSAS; Liebowitz, 1987) was included as a measure of social anxiety. As the LSAS was developed for use in adults, participants aged 17 and under will complete the Liebowitz Social Anxiety Scale for Children and Adolescents (LSAS-CA; Masia-Warner et al., 2003). Both scales consist of 24-items inquiring into the degree to which social anxiety plays a role in participants’ lives in everyday situations. Each item depicts a specific situation, and participants are asked to rate their level of fear and avoidance of that situation. Fear is measured on a 4-point Likert scale from 0 (None) to 3 (Severe) and avoidance is measured on a 4-point Likert scale from 0 (Never) to 3 (Usually). An example item from the LSAS is: “Going to a party”. An example item from the LSAS-CA is: “Going to parties, dances, or school activities.” Both the adult and adolescent versions of the scale demonstrate good psychometric properties (Baker et al., 2002; Masia-Warner et al., 2003). Higher scores on this scale are indicative of greater levels of social anxiety.

***Social Connectedness Scale – Revised***

The Revised Social Connectedness Scale (SCS-R; Lee et al., 2001) was included as a measure of social connectedness. The scale consists of 20 items, 10 positively worded and 10 negatively worded, enquiring into the extent to which individuals feel connected to others in their social environment. Items are rated on a 6-point Likert scale from 1 (Strongly disagree) to 6 (Strongly agree). An example item is: “I feel close to people”. The scale is a well-established social connectedness instrument (Kelley et al., 2025) and has demonstrated good psychometric properties in both adolescent and adult samples (e.g., Capanna et al., 2013; Too et al., 2022). Higher scores on this scale indicate greater social connectedness.

**References**

Baker, S. L., Heinrichs, N., Kim, H.-J., & Hofmann, S. G. (2002). The Liebowitz social anxiety scale as a self-report instrument: A preliminary psychometric analysis. *Behaviour Research and Therapy, 40*(6), 701–715. [https://doi.org/10.1016/S0005-7967(01)00060-2](https://psycnet.apa.org/doi/10.1016/S0005-7967(01)00060-2)

Capanna, C., Stratta, P., Collazzoni, A., D'Ubaldo, V., Pacifico, R., Di Emidio, G., Ragusa, M., & Rossi, A. (2013). Social connectedness as resource of resilience: Italian validation of the Social Connectedness Scale—Revised. *Journal of Psychopathology, 19*(4), 320–326.

Kelley, B., Fraser, B., Wells, A., & Ferdock, M. (2025). Psychometric analysis of the social connectedness instrument. *Frontiers in Psychology*, *16*, 1565267. <https://doi.org/10.3389/fpsyg.2025.1565267>

Lee, R. M., Draper, M., & Lee, S. (2001). *Social Connectedness Scale--Revised (SCS-R)* [Database record]. APA PsycTests. <https://doi.org/10.1037/t16389-000>

Liebowitz, M. R. (1987). Social phobia. *Modern Problems of Pharmacopsychiatry*, *22*, 141-173. <https://doi.org/10.1159/000414022>

Masia-Warner, C., Storch, E. A., Pincus, D. B., Klein, R. G., Heimberg, R. G., & Liebowitz, M. R. (2003). The Liebowitz social anxiety scale for children and adolescents: an initial psychometric investigation. *Journal of the American Academy of Child and Adolescent Psychiatry, 42*(9), 1076–1084. <https://doi.org/10.1097/01.CHI.0000070249.24125.89>

Too, E. K., Chongwo, E., Mabrouk, A., & Abubakar, A. (2022). Adolescent Connectedness: A Scoping Review of Available Measures and Their Psychometric Properties. *Frontiers in Psychology, 13*, 856621. <https://doi.org/10.3389/fpsyg.2022.856621>
